# Dual fluorescent aptasensor for simultanous and quantitative detection of sulfadimethoxine and oxytetracycin residues in animal-derived foods tissues based on mesoporous silica

**DOI:** 10.3389/fnut.2022.1077893

**Published:** 2022-12-21

**Authors:** Jiaming Tang, Xiaoling Zheng, Shuang Jiang, Mingdong Cao, Sixian Wang, Zhaoyang Zhou, Xunqing Nie, Yu Fang, Tao Le

**Affiliations:** College of Life Sciences, Chongqing Normal University, Chongqing, China

**Keywords:** fluorescent aptasensor, sulfadimethoxine, oxytetracycline, silica nanoparticles, quantitative detection

## Abstract

Herein, we developed a dual fluorescent aptasensor based on mesoporous silica to simultaneously detect sulfadimethoxine (SDM) and oxytetracycline (OTC) in animal-derived foods. We immobilized two types of aptamers modified with FAM and CY5 on the silica surface by base complementary pairing reaction with the cDNA modified with a carboxyl group and finally formed the aptasensor detection platform. Under optimal conditions, the detection range of the aptasensor for SDM and OTC was 3–150 ng/mL (*R*^2^ = 0.9831) and 5–220 ng/mL (*R*^2^ = 0.9884), respectively. The limits of detection for SDM and OTC were 2.2 and 1.23 ng/mL, respectively. The limits of quantification for SDM and OTC were 7.3 and 4.1 ng/mL, respectively. Additionally, the aptasensor was used to analyze spiked samples. The average recovery rates ranged from 91.75 to 114.65% for SDM and 89.66 to 108.94% for OTC, and all coefficients of variation were below 15%. Finally, the performance and practicability of our aptasensor were confirmed by HPLC, demonstrating good consistency. In summary, this study was the first to use the mesoporous silica-mediated fluorescence aptasensor for simultaneous detection of SDM and OTC, offering a new possibility to analyze other antibiotics, biotoxins, and biomolecules.

## 1 Introduction

Farmers often use sulfadimethoxine (SDM) and oxytetracycline (OTC) as antimicrobial treatments for animals due to their low cost and high efficacy (1, 2). However, some studies have shown that SDM and OTC residues in animals enter the human body through the food chain, causing allergies, renal failure, liver function impairment, and other hazards to the body (3, 4). Therefore, the European Commission and China set the maximum residue level of SDM and OTC in food to 100 ng/mL (5, 6). At present, the methods of detecting antibiotic residues usually include high-performance liquid chromatography (HPLC) (7), HPLC coupled with mass spectrometry (HPLC-MS) (8), enzyme-linked immunoassay (ELISA) (9), and capillary electrophoresis (10). These methods have the advantages of high efficiency, sensitivity, and specificity. However, their disadvantages are evident. HPLC and HPLC-MS can only detect one sample at a time and are not suitable for mass detection of samples. Moreover, the antibodies required by ELISA are highly affected by the environment, and the capillary electrophoresis equipment is costly. Therefore, developing a novel high-throughput, low-cost, and stable detection method for antibiotic residues in animal-derived foods is imperative.

Sensors based on aptamers as signal probes are of particular interest due to their good selectivity for target molecules and stability in complex physicochemical environments (11). Recently, various aptasensors have been used to detect SDM and OTC residues, such as chemiluminescence (12, 13), electrochemical (14, 15), colorimetric (16, 17), fluorescence (18, 19). These methods have the advantages of high sensitivity and specificity but can only be used to detect a single target. Therefore, Díaz-García et al. created a gold-based colorimetric assay to simultaneously detect SDM and OTC residues in milk (20). However, due to the physical and chemical properties of gold nanoparticles, which are easily affected by the surrounding detection environment, this method has shortcomings such as poor reproducibility and inconsistent detection results. Therefore, constructing a new multiplexed aptasensor to detect SDM and OTC residues in animal-derived foods is urgent.

Fluorescent aptasensors based on porous materials (carbon nanotubes, mesoporous silica, graphene oxide, etc.) are widely used for the monitoring of contaminants in food because of their high specificity and high accuracy (21–23). Among them, silicon nanoparticles have the advantages of simple preparation, easy surface modification, non-toxicity and good thermal stability, and are widely used to construct aptasensors to detect antibiotic residues (24–26). However, most studies have relied on mesoporous silica interacting with aptamers to create molecular gates, isolating fluorescein molecules in the pores and finally releasing the fluorescein molecules by target-specific binding to the corresponding aptamers. These methods depend on the pore size (27, 28). If the pore size is too large, the sealing of the molecular gate will be poor. If it is too small, it will be difficult for the fluorescein molecules to enter the molecular gate. This effect increases the difficulty of preparing mesoporous silica (29, 30). Hence, using the easy modification of the surface of mesoporous silica to immobilize fluorescein molecules on the silica surface can effectively avoid the loss of fluorescein molecules and improve the sensor’s accuracy.

Based on these ideas, we proposed for the first time a dual fluorescent signal aptasensor based on aminoaminated mesoporous silica (MSN-NH_2_) to simultaneously detect SDM and OTC residues in animal-derived foods. First, we used MSN-NH_2_ as a loading platform for aptamers. Then, the fluorophore-modified aptamer was attached to silica using the carboxyl-modified cDNA as a linker. Finally, the aptamers were released from the silica surface when the test sample contained SDM and OTC. The quantitative detection of SDM and OTC was realized according to the fluorescence intensity in the supernatant. The whole detection process was efficient and sensitive, providing a broad prospect for quantitative detection of SDM and OTC.

## 2 Experimental

### 2.1 Materials and apparatus

The SDM, OTC, sulfadiazine, sulfamethoxypyridazine, kanamycin, doxycycline, 3-amino-propyltriethoxysilane, hexadecyl trimethyl ammonium bromide (CTAB), Tetraethyl orthosilicate (TEOs), and other chemical reagents were purchased from Aladdin Co., Ltd., (Shanghai, China).

The characterization images of MSN and MSN-NH_2_ particle size were obtained from JEM-2100F (Japan). The amino modification success of mesoporous silica was verified by Spectrum 100 (PerkinElmer, USA) and UV-2450 (Shimadzu, Kyoto, Japan). The accuracy of the aptasensor was confirmed by HPLC (Ultimate 3000).

All the nucleotide sequences below were synthesized by Sangong Biotechnology Co., Ltd., (Shanghai, China).

Sulfadimethoxine (SDM) aptamer 5′FAM-GAGGGCAACG AGTGTTTATAGA-3′, (31).

Oxytetracycline (OTC) aptamer 5′-GAGCCGGGCGCGGT ACGGGTACTGGTA-CY5-3′ (32).

Linker: 5′COOH-TCTATAAACACACTCGTTGCCCTCT TTTTTTCTCGGCCCGCGCCATGCCCATGACCAT-3′

### 2.2 Synthesis of MSN

The MSN composite was synthesized based on previous reports with minor modifications (24, 27, 33). Briefly, 1.0 g of CTAB was dissolved in 480 mL of deionized water and stirred slowly at 80°C until a clear solution was obtained. Then, we added 3.5 mL NaOH (2M) and 5.0 mL tetraethyl orthosilicate to the above mixture dropwise, stirred vigorously for 2 h, and cooled to room temperature. Finally, the white precipitate was centrifuged at 15,000 rpm for 10 min and successively washed three times with ethanol and water to remove any solvent remaining in the pores. The washed products were dried under a vacuum overnight at 60°C to obtain MSNs containing CTAB templates. The prepared MSNs were cauterized at a high temperature for a long time to remove unreacted CTAB.

### 2.3 Synthesis of MSN-NH_2_

First, 0.5 g of MSNs were ultrasonically dispersed in 100 mL of ethanol, then 2.5 mL of 3-amino-propyltriethoxysilane was added. The mixture was stirred slowly and uniformly at room temperature for 24 h. Next, the suspension was centrifuged at 8,000 rpm for 15 min, and the MSN-NH_2_ was washed three times with ethanol and ultrapure water to remove excess 3-amino-propyltriethoxysilane. The product was vacuum-dried overnight at 60°C. Finally, the dry white MSN-NH_2_ powder was stored at room temperature for later use.

### 2.4 Preparation of dual fluorescent aptasensors

First, 40 mg of MSN-NH_2_ was sonicated in 500 μL phosphate buffer (PBS, 0.1 M, PH = 7.4) for 5 min to disperse uniformly. To form the MSN-NH_2_/cDNA complex, 70 μL of cDNA was added to the above mixture and incubated for 3 h at 25°C with shaking. The reacted solution was centrifuged at 15,000 rpm for 5 min, and the MSN-NH_2_/cDNA was washed with PBS to remove excess cDNA. Subsequently, 500 μL of PBS was added again to form an MSN-NH_2_/cDNA complex suspension. Next, 50 μL of SDM (15 μM) FAM-aptamer and 75 μL of OTC (10 μM) CY5-aptamer were added, and the cells were incubated with shaking in the dark for 1 h. It was centrifuged again, the pellet was washed with PBS, and the MSN-NH_2_/cDNA/aptamer complex was formed. Finally, the obtained pellet was suspended in PBS and stored at 4°C for further use.

The linear relationship between the target concentration and the fluorescence intensity of the aptasensor was established as follows: 5 μL of the above particle suspension was added to 200 μL of PBS with different concentrations of SDM (0, 4.6875, 9.375, 18.75, 37.5, 75, and 150 ng/mL) and OTC (0, 5.625, 11.25 22.5, 55, 110, and 220 ng/mL). It was then reacted in the dark at 25°C for 1 h. Finally, the reaction mixture was centrifuged at 15,000 rpm for 5 min, and the supernatant was retained. Correlations between the fluorescence intensity in the supernatant and SDM and OTC concentrations were calculated using ORIGIN (FAM: λ_ex_ = 492 nm and λ_em_ = 518 nm; CY5: λ_ex_ = 649 nm and λ_em_ = 670 nm). The limit of detection (LOD) of the dual fluorescent aptasensor was calculated according to the equation: LOD = 3SD/slope. The SD represented the standard deviation of the fluorescence intensity in the control group. The slope comprised the linear relationship between the fluorescence intensity of the target and the dual fluorescence aptasensor at different concentrations (11).

### 2.5 Dual fluorescence aptasensors analyze real samples

The practical application capability of the aptasensor was verified by adding known concentrations (50, 100, and 150 ng/mL) of SDM and OTC to milk and honey. Milk and honey were purchased from local supermarkets and verified to be free of SDM and OTC by HPLC. Sample pretreatment was performed as previously described (34–37). Recovery and coefficient of variation were chosen as performance parameters for the aptasensor and were validated by HPLC. The recovery formula was: (measured concentration/added concentration) x 100%. Each concentration was measured five times (12).

## 3 Results and discussion

### 3.1 Principle of dual fluorescence aptasensors

**FIGURE 1:**
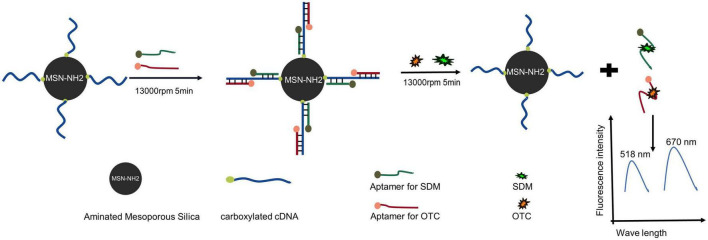
Schematic diagram of a dual fluorescent aptasensor based on MSN-NH_2_ to detect sulfadimethoxine (SDM) and oxytetracycline (OTC).

The schematic diagram of the silica-based dual fluorescent aptasensor for simultaneous detection of SDM and OTC is shown in Figure 1. The carboxyl-modified cDNA was immobilized on the silica surface by forming an amide bond with MSN-NH_2_. The SDM and OTC aptamers, labeled with FAM and CY5, respectively, were used as signaling probes, captured by cDNA *via* the Watson-Crick base pairing principle, and immobilized on the silica surface. Finally, a dual-fluorescent aptasensor with MSN-NH_2_/cDNA/aptamer as a composite material was formed. In the presence of SDM and OTC, the aptamer was released into the supernatant, resulting in decreased fluorescence of the aptasensor detection platform and increased fluorescence in the supernatant. The final fluorescence value in the supernatant was linearly correlated with the target concentration after centrifugation, resulting in the detection of SDM and OTC.

### 3.2 Characterization of MSN-NH_2_

**FIGURE 2:**
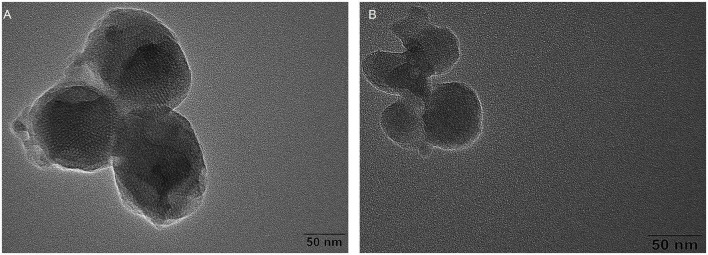
**(A)** Transmission electron microscope (TEM) micrograph of MSN; **(B)** TEM micrograph of MSN-NH_2_.

**FIGURE 3:**
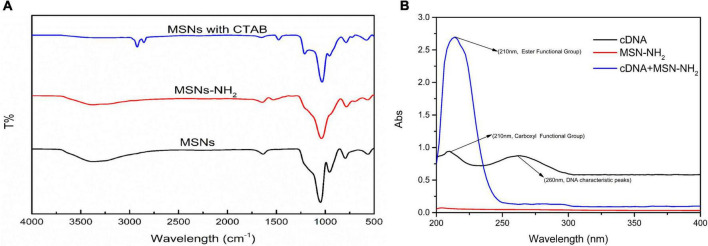
**(A)** FTIR spectra of MSN-NH_2_, cDNA, and cDNA/MSN-NH_2_; **(B)** UV-Vis spectra of supernatant of pure aptamer, aptamer/MSN, and aptamer/MSN-NH_2_ complexes.

The transmission electron microscope (TEM) images of MSN and MSN-NH_2_ are presented in Figures 2A, B. The average size of the synthesized MSN and MSN-NH_2_ was 50 nm. The FTIR spectra of MSN, MSN-CTAB, and MSN-NH_2_ are shown in Figure 3A. The blue line shows distinct absorption peaks at 2,921 and 2,851 cm^–1^ due to the residual CTAB on the surface of MSN (27). After high-temperature burning, the black line has no clear peak in this band, indicating that CTAB was removed. After adding APTES, the red line appeared at 1,636 and 1,554 cm^–1^, representing the bending stretching vibration of amide and the characteristic peaks of NH_2_, respectively, which indicated that the amino group was modified on the surface of MSN. Additionally, the carboxyl group-modified cDNA has a carboxyl group characteristic peak at 210 nm, and the MSN-NH_2_/cDNA combination produces an amide bond, resulting in a significant increase in the absorbance at 210 nm, which also indicates that the amino group of MSN was successfully modified (Figure 3B).

### 3.3 Optimization of dual fluorescence aptasensor performance

To improve the performance of the dual fluorescent aptasensor, we optimized the aptamer concentration, aptamer/target incubation time, and cDNA/aptamer reaction time based on the fluorescence intensity. Besides, to verify the influence of SDM and OTC aptamers on the sensor performance, all optimization conditions were divided into incubating aptamers simultaneously or alone with MSN-NH_2_/cDNA.

**FIGURE 4:**
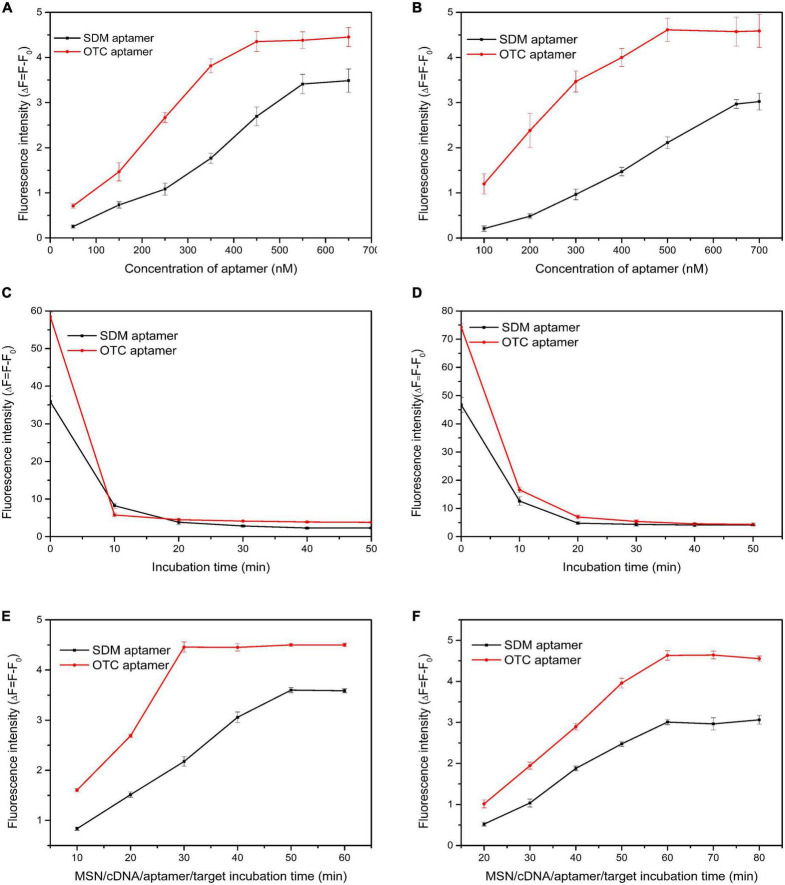
**(A,B)** Optimization of aptamer concentration; **(C,D)** optimization of incubation time of aptamer/cDNA; **(E,F)** optimization of incubation time for aptamer/target. The concentrations of sulfadimethoxine (SDM) and oxytetracycline (OTC) were set at 100 ng/mL. (Panels **A,C,E**: SDM and OTC aptamers were added separately; panels **B,D,F**: SDM and OTC aptamers were added simultaneously).

Aptamer concentrations varied from 100 to 700 nM. When the two tapes of aptamers reacted with the MSN-NH_2_/cDNA complex alone, the fluorescence intensity reached the maximum at 450 and 550 nM (Figure 4A). When the two tapes of aptamers were simultaneously complexed with MSN-NH_2_/cDNA, the aptamer concentrations corresponding to the maximum fluorescence intensity increased by 50 and 100 nM, respectively (Figure 4B). These results indicated that, with increasing aptamer concentrations, the competitiveness of the two aptamers with MSN-NH_2_/cDNA complexes gradually increased.

The aptamer/cDNA incubation time varied from 0 to 50 min. The aptamer reacted most rapidly with cDNA within 10 min, and the fluorescence value changed the fastest (Figures 4C, D). From 10 to 20 min, the fluorescence value did not change much (Figure 4C), indicating that the reaction of the two aptamers alone with the MSN-NH_2_/cDNA complex was saturated at 10 min. When the aptamers reacted simultaneously with the MSN-NH_2_/cDNA complex, they tended to be stable after 20 min (Figure 4D).

The aptamer/target incubation time varied from 10 to 80 min. As shown in Figure 4E, when the two types of aptamers were incubated with MSN-NH_2_/cDNA alone, the OTC and SDM aptamers fully reacted with the target at 30 and 50 min, respectively. As shown in Figure 4F, when the two types of aptamers were incubated with MSN-NH_2_/cDNA at the same time, the fluorescence intensity reached the maximum at 60 min, and the maximum fluorescence value also decreased accordingly. These results indicated that when aptamers are present simultaneously, they will affect each other to a certain extent.

### 3.4 Qualitative determination of SDM and OTC by dual fluorescent aptasensors

**FIGURE 5:**
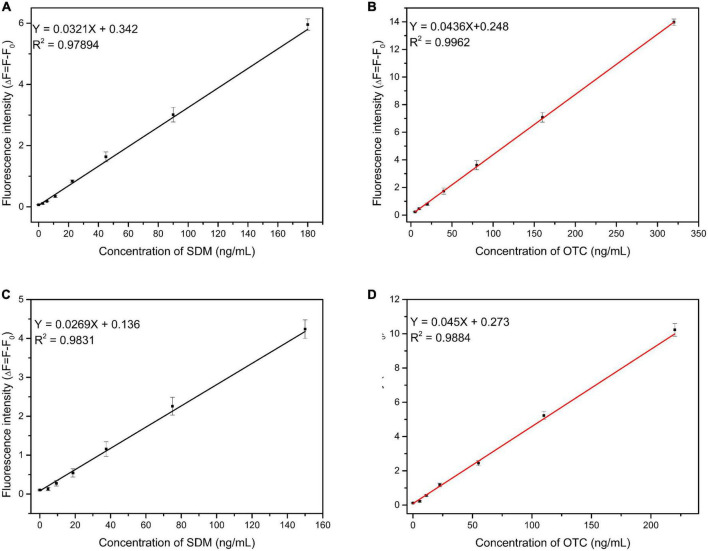
Linearity between fluorescence intensity and target concentration. **(A,B)**: Two types of aptamers were added separately; **(C,D)**: two types of aptamers were added together. (The concentrations of sulfadimethoxine (SDM) and oxytetracycline (OTC) were set at 100 ng/mL).

Under optimized experimental conditions, SDM and OTC were simultaneously detected with the proposed method. The limits of detection and linear ranges of the two aptamers were also verified when added individually in order to exclude the effect of competition on experimental precision when both aptamers were added simultaneously. When the two types of aptamers were added alone, the linear relationship between different OTC and SDM concentrations and the fluorescence value are shown in Figures 5A, B: *y* = 0.0436x + 0.248 (OTC 2.5–320 ng/mL), *y* = 0.0321x + 0.342 (SDM 2.5–180 ng/mL). The LOD was 3.81 ng/mL for OTC and 0.64 ng/mL for SDM. When the two types of aptamers were added simultaneously, the increase in target concentration and the linear change of its fluorescence value are shown in Figures 5C, D: *y* = 0.045x + 0.273 (OTC: 5–220 ng/mL), *y* = 0.0269x + 0.136 (SDM: 3–150 ng/mL). The LOD was 1.23 ng/mL for OTC and 2.2 ng/mL for SDM. The limits of quantification for SDM and OTC were 7.3 and 4.1 ng/mL, respectively. These results showed that the different addition methods of aptamers did not affect the accuracy of system.

**FIGURE 6:**
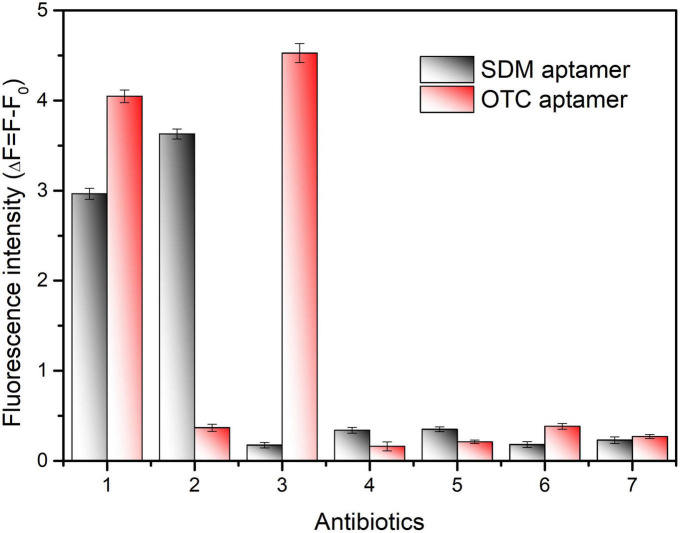
Specificity validation of the dual fluorescent aptasensor for sulfadimethoxine (SDM) and oxytetracycline (OTC). The concentration of all antibiotics was set to 100 ng/mL. (1) SDM + OTC, (2) SDM, (3) OTC, (4) Sulfamethoxypyridazine, (5) Sulfadiazine, (6) Doxycycline, (7) Kanamycin.

In additation, several antibiotics at 100 ng/mL were used to evaluate the specificity of the proposed method to detect SDM and OTC. As shown in Figure 6, although the fluorescence values change significantly in the separate and mixed systems, the aptasensor remains significantly specific for SDM and OTC compared to several of the remaining antibiotics. Furthermore, the fluorescence values of the mixture of SDM and OTC did not change much from that of the individual systems, indicating that SDM and OTC do not interact. Therefore, this method has great application prospects for the simultaneous detection of SDM and OTC in foods.

### 3.5 Validation of dual fluorescent aptasensors

In order to verify the detection effect of real samples, the proposed method was applied to the determination in milk and honey samples. As shown in Table 1, the recoveries of SDM and OTC in milk were 92.32–106.53%, 98.81–107.82%, respectively; the recoveries of SDM and OTC in honey were 91.75–114.65%, 89.66–108.94%, respectively. The relative standard deviation for detecting SDM and OTC ranged from 4.89 to 14.65% and 3.78 to 11.12%, respectively. The accuracy and precision of the method were verified to be within acceptable limits. As shown in Table 2, the proposed method exhibits higher sensitivity, and a wider linear detection range than previous methods for the detection of SDM and OTC. Additionally, the detection results of this method were positively correlated with the HPLC results, indicating that the proposed method is reliable for the simultaneous detection of SDM and OTC.

**TABLE 1:** A brief overview of recently reported methods for the determination of sulfadimethoxine (SDM) and oxytetracycline (OTC).

Analyte	Sample	HPLC			Aptasensor	
		**Spiked amount (ng/mL)**	**Mean recoveries (%)**	**RSD (%)**	**Mean recoveries (%)**	**RSD (%)**
SDM	Milk	50	98.30	1.61	95.70	4.89
		100	99.62	4.27	106.53	6.24
		150	102.78	3.48	92.32	12.35
	Honey	50	98.84	1.37	97.52	5.68
		100	98.13	2.41	114.65	10.26
		150	99.08	3.13	91.75	14.65
OTC	Milk	50	103.06	1.52	107.08	3.78
		100	100.68	2.31	98.21	5.19
		150	98.35	2.76	94.92	11.64
	Honey	50	101.36	1.96	105.9	3.26
		100	103.21	2.60	108.94	9.81
		150	101.89	3.71	89.66	11.12

**TABLE 2:** Recoveries and coefficients of variation of spiked samples and comparison between aptasensor and high performance liquid chromat ography (HPLC) results (*n* = 5).

	Analytical methods	LOD (ng/mL)	Detection range (ng/mL)	References
SDM	HPLC	2	10–2,000	(38)
	ELISA	1.86	2.42–7.74	(39)
	Fluorescence aptasensor	3.41	2–300	(3)
	In this work	2.2	3–150	/
OTC	HPLC	5	20–1,000	(40)
	ELISA	15	1.02–128.46	(41)
	Fluorescence aptasensor	0.874	4.604–230.23	(42)
	In this work	1.23	5–220	/

## 4 Conclusion

Herein, we established a novel dual fluorescent aptasensor based on MSN-NH_2_ for simultaneous detection of SDM and OTC in edible tissues. The two types of aptamers were labeled with FAM and CY5 fluorophores, respectively. SDM and OTC competed with the cDNA for the corresponding aptamer to make the fluorophore off the silica surface to achieve the quantitative target detection according to the fluorescence intensity in the supernatant. In addition, the accuracy of the proposed method is ensured whether the aptamers are added alone or simultaneously. Finally, the proposed method demonstrated excellent performance in real sample assays with high recoveries and good coefficients of variation. To the best of our knowledge, a strategy based on the immobilization of two aptamers on a silica surface by cDNA has not been reported. The rational design of this method comprises a strategy to simultaneously detect multiple antibiotic residues in food, laying the foundation for future aptasensor detection of multiple targets.

## Data availability statement

The original contributions presented in this study are included in the article/supplementary material, further inquiries can be directed to the corresponding author.

## Author contributions

JT: conceptualization, methodology, and writing. XZ: methodology, validation, investigation, and writing. SJ: methodology, investigation, and reviewed the manuscript. MC, SW, ZZ, XN, and YF: methodology, validation, and investigation. TL: supervision and project administration. All authors have contributed to the article, read, and agreed to the published version of the manuscript.
